# Long-Term Host Immune Modulation Following Tisagenlecleucel Administration in Patients with Diffuse Large B-Cell Lymphoma and B-Lineage Acute Lymphoblastic Leukemia

**DOI:** 10.3390/cancers15092411

**Published:** 2023-04-22

**Authors:** Anna Guarini, Giulia Radice, Nadia Peragine, Chiara Buracchi, Maria Stefania De Propris, Alice Di Rocco, Arianna Di Rocco, Sabina Chiaretti, Alex Moretti, Sara Napolitano, Maurizio Martelli, Adriana Balduzzi, Giuseppe Gaipa, Andrea Biondi, Robin Foà

**Affiliations:** 1 Hematology, Department of Translational and Precision Medicine, Sapienza University, 00185 Rome, Italy; 2 Tettamanti Center, Fondazione IRCCS San Gerardo dei Tintori, 20900 Monza, Italy; 3 Department of Public Health and Infectious Diseases, Sapienza University, 00185 Rome, Italy; 4 Pediatrics, Fondazione IRCCS San Gerardo dei Tintori, 20900 Monza, Italy; 5 School of Medicine and Surgery, University of Milano-Bicocca, 20126 Milan, Italy

**Keywords:** CAR-T cells, DLBCL, B-ALL, immune modulation

## Abstract

**Simple Summary:**

We hereby report on the immunomodulatory effects induced over time in patients with advanced relapsed or refractory (R/R) diffuse large B-cell lymphoma (DLBCL) and B-lineage acute lymphoblastic leukemia (B-ALL) treated with the commercial CAR-T-cell product tisagenlecleucel. We observed that, in addition to its anti-neoplastic activity, tisagenlecleucel was capable of exerting a marked and long-lived in vivo reshaping of the host immune system, in terms of T- and NK-cell expansion and cytokine release. These results may contribute to a further understanding of the complex field of immunotherapy and intercellular crosstalk, suggesting a role for host immune modulation in the long-term control of the disease.

**Abstract:**

**Background:** Chimeric antigen receptor (CAR)-T cells represent a potentially curative strategy for patients with relapsed or refractory (R/R) B-cell malignancies. To elucidate a possible host immune activation following CAR-T-cell infusion, we investigated the effects of tisagenlecleucel administration on the patients’ immune populations in 25 patients with R/R diffuse large B-cell lymphoma (DLBCL) and B-lineage acute lymphoblastic leukemia (B-ALL). **Methods:** The modulation of CAR-T cells over time, the numeric changes, as well as the cytokine production capability of different lymphocyte populations and circulating cytokine levels, were analyzed. **Results:** Our results confirmed the ability of tisagenlecleucel to control the disease, with an overall response observed in 84.6% of DLBCL and in 91.7% of B-ALL patients at 1-month post-infusion, and showed that most patients who subsequently relapsed could undergo further treatment. Interestingly, we could document a significant increase in CD3^+^, CD4^+^, CD8^+^, and NK cells over time, as well as a decrease in Treg cells, and an increased IFNγ and TNFα production by T lymphocytes. **Conclusions:** Taken together, our results indicate that in patients with DLBCL and B-ALL, the administration of tisagenlecleucel is capable of inducing a marked and prolonged in vivo modulation/reshaping of the host immune system, both in children and adults.

## 1. Introduction

Harnessing the host immunity has proven a promising approach to specifically target tumor cells. The well-known anti-tumor activity of T lymphocytes has been indeed at the core of numerous therapeutic strategies, the most recent and encouraging of which is represented by chimeric antigen receptor (CAR)-T cells [1,2]. CAR-T cells are T lymphocytes, mostly autologous, genetically engineered ex vivo to express artificial receptors specific for a tumor-related antigen and capable of binding and lysing neoplastic cells [3]. CAR-T-cell therapy has been associated with important clinical results in the management of different hematological B-cell malignancies, primarily due to the targeting of CD19, a transmembrane protein whose expression pattern is restricted to the B-cell lineage and is present at all differentiation stages. Tisagenlecleucel, a second-generation CAR-T-cell product, targets B cells through the FMC63 ScFv and activates the engineered T cells via intracellular signaling domains, combining a CD3-ζ domain with the 4-1BB co-stimulatory domain.

CAR-T cells have been initially approved for the treatment of relapsed/refractory (R/R) children and young adults with B-lineage acute lymphoblastic leukemia (B-ALL) and then for R/R diffuse large B-cell lymphoma (DLBCL) adult patients [4,5,6]. The approval of the second-generation CAR-T-cell product tisagenlecleucel was based on the favorable results of the ELIANA study on 75 pediatric and young adult R/R B-ALL [4] and of the JULIET trial on 93 R/R DLBCL patients [5]. Following these pivotal studies, there have been numerous subsequent reports confirming the validity of this innovative strategy [7,8], and CAR-T cells have also been approved for different forms of non-Hodgkin’s lymphoma [9,10] and recently for R/R multiple myeloma [11,12]. The field is continuously evolving with attempts to develop more effective CAR-T cells, as well as more refined CAR-T-cell products and more efficient and rapid manufacturing [13,14].

While the clinical application of CAR-T cells is progressively extending to more diseases and indications, very little is known about the immune modulation induced in vivo in patients treated with CAR-T cells. Based on preclinical models, it has been suggested that CAR-T cells, in addition to targeting cancer cells, may also activate the host immune system [15], and the possible role of an in vivo production of interferon-gamma (IFNγ) has also been hypothesized in a syngeneic mouse glioblastoma model [16].

In order to further elucidate a possible host immune activation following the infusion of CAR-T cells, in this study, we investigated the changes induced in vivo in 25 patients with B-ALL and DLBCL treated with tisagenlecleucel. To this end, we monitored the modulation of CAR-T cells over time and assessed the changes in T- and NK-cell distribution, as well as the cytokine production capability by different lymphocyte populations and circulating cytokine levels. The obtained results clearly document that, in patients with B-ALL and DLBCL, the administration of tisagenlecleucel is capable of inducing a marked in vivo crosstalk that leads to a profound modulation/reshaping of the host immune system over time, both in children and adults.

## 2. Materials and Methods

### 2.1. Patients

This observational prospective study was carried out on a cohort of 25 patients admitted to the Hematology Center at the Sapienza University of Rome (15 patients: 13 DLBCL and 2 B-ALL) and the Department of Pediatrics of Monza (10 B-ALL patients). In terms of the inclusion criteria, all DLBCL patients were R/R after a median of 2 previous lines of therapy (range 2–4), 2 of whom had also undergone an autologous stem cell transplant, and all were ineligible for further treatment. B-ALL patients were R/R after a median of 3 lines of treatment (range 2–6), 5 of whom had undergone an allogeneic transplant. For B-ALL patients, indications for treatment comprised an age limit of 25 years (as per indication), while for DLBCL patients, the age limit in Italy was initially 70 and then 75 years.

Treatment consisted of one infusion with the commercial second-generation CD19 CAR-T-cell product tisagenlecleucel (Kymriah™). The dosage of CAR-T cells administered to each patient is reported in Supplementary Table S1. The first step in CAR-T-cell production was apheresis for the lymphocyte collection; cells were then shipped to the manufacturing facility, where they were engineered to express the transgene of the chimeric receptor and then expanded and shipped back to the clinical site. Subsequently, patients underwent lymphodepleting chemotherapy, consisting of fludarabine 30 mg/m^2^ administered daily for 3 days for DLBCL patients and fludarabine for 4 days and cyclophosphamide for 2 days for B-ALL patients, followed by the infusion of CAR-T cells.

B-ALL patients included 4 females and 8 males, with a median age of 10 years (range 3–21), while DLBCL patients included 5 females and 8 males, with a median age of 55 years (range 34–71).

The mean percentage of blast cells in B-ALL patients prior to lymphodepleting chemotherapy was 32% ± 50 (range 0–90%). The mean follow-up period at data cutoff after the infusion was 16.9 months (range 2.7–24.5) for B-ALL and 11.5 months (range 3–28.3) for DLBCL patients. Samples from B-ALL and DLBCL patients were obtained with informed consent and ethical approval in accordance with the Declaration of Helsinki and the Ethics Committee of the two institutions (Rome and Monza). Patients’ characteristics are summarized in Supplementary Table S1. 

### 2.2. Immunophenotyping

Peripheral blood (PB) lymphocytes were obtained from whole-blood samples collected on day 0 before CD19 CAR-T-cell infusion and at multiple time points after the infusion (days 3, 7, 14, and 28; months 3 and 6), following the EuroFlow Bulk Lysis protocol [17]. Flow cytometry was performed using an 8-color monoclonal antibody (moAb) combination to monitor CAR-T cells and lymphocyte subsets (T, B, and NK cells). CAR^+^ cells were assessed following incubation, with the biotinylated CD19 antigen and FITC- or APC-conjugated anti-biotin protein, according to their manufacturers’ instructions (Acro Biosystems, Beijing, China, and Miltenyi Biotec, Bergisch Gladbach, Germany, respectively). Lymphocyte subsets (T, B, and NK cells) were analyzed via staining with a combination of labeled moAbs against the CD3, CD4, CD8, CD16, CD19, CD45, CD56, and CD62L antigens; for Treg cells, moAbs against tCD39, HLADR, CD3, CD45RA, CD127, CD4, CD25, and CD45 were used. All antibodies were from Becton Dickinson (BD, San Jose, CA, USA). The samples were acquired using the FACSCanto II flow, collecting at least 30,000 events, and analyzed using the Paint-A-Gate software (BD, San Jose, CA, USA). The representative plots of the flow gating strategy are reported in Supplementary Figure S1.

Absolute cell counts were determined using the correlation of the percentage of positive populations to WBC. The total WBC counts and lymphocyte percentages for DLBCL and B-ALL patients at the different time points studied (T0–T6M) are reported in Supplementary Figure S2.

### 2.3. Intracellular Cytokine Production by T and NK Cells

Peripheral blood mononuclear cells (PBMCs) were obtained from whole-blood samples collected before the CAR-T-cell infusion and at different time points after the infusion as detailed above, via Ficoll–Paque density centrifugation (Nycomed Pharma AS, Oslo, Norway). To assess cytokine production, fresh, isolated PBMCs were cultured in a complete RPMI 1640 medium (Cambrex BioScience, Verviers, Belgium) supplemented with 10% heat-inactivated fetal bovine serum (FBS, HyClone, South Logan, UT, USA), 0.3 mg/mL L-glutamine, and 1% Pen-strep (Euro-Clone, Pero, Italy) for 4 h in the presence of phorbol 12-myristate 13-acetate 25 ng/mL (PMA; Sigma-Aldrich, St. Louis, MO, USA) and ionomycin 1 μg/mL (Iono; Sigma-Aldrich) for cell activation, and a GolgiStop™ Protein Transport Inhibitor solution (BD) for cytokine secretion inhibition. Activated T cells were washed twice in PBS + 0.2% FBS, divided into tubes, and then fixed and permeabilized with a Fixation/Permeabilization Kit (BD), according to the manufacturer’s instructions. For T- and NK-cell cytokine production, PBMCs were stained with FITC-, PE-, PerCP-, PE-Cy7-, and APC-labeled moAbs against CD3, CD4, CD16, CD56, IFNγ, and tumor necrosis factor-alpha (TNFα). After washing, the samples were acquired using a FACSCanto I flow cytometer (BD) and analyzed using the FACSDIVA software (BD). The representative plots of the flow gating strategy are reported in Supplementary Figure S3.

Absolute cell counts were determined by correlating the percentage of cytokine-producing populations to the total lymphocyte numbers.

### 2.4. Plasma Cytokine Determination

For plasma collection before and at different time points after CAR-T-cell infusion, PB samples were centrifuged at 3000 rpm at 20 °C for 10 min, and the supernatants were carefully harvested and stored at −80 °C until cytokine analyses. Concentrations of IL-1β, IL-2, IL-4, IL-6, IL-8, IL-10, IL-12p70, IL-17A, IFNγ, and TNFα in the plasma were quantitatively determined using the Cytometric Bead Array (CBA) technique (CBA Human Th1/Th2/Th17 Cytokine Kit, CBA Human Inflammatory Cytokine Kit, and CBA Flex Set; BD) according to the manufacturer’s instructions. Following the acquisition of the samples on the FACSCanto I flow cytometer (BD), the results were generated in a graphical and tabular format using the BD CBA software FCAP 3.0.1 (BD).

### 2.5. Statistical Analysis

The 2-sided Student’s *t*-test was used to evaluate the significance of differences between the groups. The results are expressed as means ± standard errors. Statistical significance was defined for a *p* value < 0.05.

## 3. Results

### 3.1. Response to Tisagenlecleucel

In the 13 patients with DLBCL, the response to CAR-T cells was assessed via PET according to the Lugano criteria [18]. At 1 month from the infusion, an overall response was observed in 11/13 patients (84.6%, 10 CR, and 1 PR), with 2 patients witnessing disease progression (PD). At 3 months, 6/13 patients (46.1%) were in CR, and 4/10 had relapsed. A late relapse (>6 months) was observed in two patients. The 8 patients who relapsed after CAR-T cells could all undergo further treatment strategies based on their good general conditions (ECOG 0; no severe cytopenia). Three patients entered an experimental protocol with a conjugated bispecific monoclonal antibody, 4 received further chemotherapy, and 2 patients underwent immunotherapy with an anti-CD79a moAb and an anti-CD19 moAb together with lenalidomide, respectively. Two patients obtained a CR and 2 achieved a PR, whereas five showed a PD. Notably, 6 of the 13 patients are alive, at a median follow-up of 17 months (Supplementary Table S1A). Regarding toxicity, 9 of the 13 patients who received CAR-T cells suffered from a cytokine release syndrome (CRS), which was grade 1 in 5 and grade 2 in 4 patients (Supplementary Table S1A). No neurologic toxicity was recorded. After a median follow-up of 11.5 months (3–28), the median OS from enrollment was 12 months (95% CI, 10 patients did not reach OS). Seven of the 13 patients have died. Of these, 2 died following a COVID-19 infection while in PR after CAR-T-cell therapy, and 1 death was related to infective complications during autologous stem cell transplantation as consolidation therapy after treatment with CAR-T cells while in CR. The other 4 patients died of PD.

In the 12 patients with B-ALL, an overall response was observed in 11/12 cases (91.7%; 8 CR and 3 PR) at 1 month from the infusion, with 1 patient experiencing a non-response. At 3 months, 6/12 patients (50.0%) were still in CR, 1 remained non-responsive, 4 patients showed a molecular relapse, and 1 patient showed disease progression. No neurologic complications were observed. Notably, 5 of the 12 patients who received CAR-T cells suffered from a CRS that was grade 4 in 2 patients, grade 2 in 1 patient, and grade 1 in 2 patients, according to the ASTCT Consensus Grading for CRF and ICANS [19] (Supplementary Table S1B). In addition, 10 of the 12 patients received further treatment, 8/10 underwent an allogeneic stem cell transplant, and 2/10 other therapies, inotuzumab in 1 patient and eldesine in the other. Nine are alive at a median follow-up of 21 months (Supplementary Table S1), with a median OS of 20 months from enrollment.

### 3.2. CAR-T-Cell Monitoring

**Figure 1:**
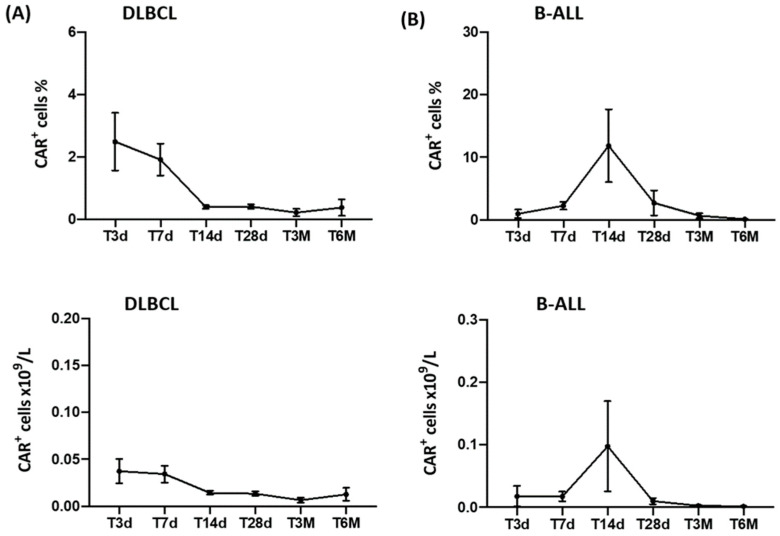
In vivo longitudinal monitoring of CD19 CAR-T cells at different time points after infusion. CAR-T cells were detected via flow cytometry up to 6 months from cell infusion. Data are reported as means ± standard errors of the percentage (upper panel) and absolute number (lower panel) of CAR^+^ cells in DLBCL (**A**) and B-ALL patients (**B**). CD19 CAR-T cells were gated within the total CD45^+^ leukocyte population.

Following CAR-T-cell infusion, we monitored the percentage and absolute number of T cells with CAR receptor expression in the peripheral blood of DLBCL and B-ALL patients over time. The trend was different in the two diseases. The percentage of CAR-T cells at T3d in DLBCL patients was equal to 2.495 ± 3.060, while at T7d, it was 1.919 ± 1.779; at T14d, it was 0.405 ± 0.210; at T28d, it was 0.412 ± 0.246; at T3M, it was 0.220 ± 0.335; and at T6M, it was 0.380 ± 0.527 (Figure 1A, upper panel). We observed a similar trend in the absolute number of CAR-T cells. At T3d, CAR-T cells (×10^9^/L) were 0.037 ± 0.043; at T7d, the number was 0.034 ± 0.031, whereas at T14d, it was 0.014 ± 0.007; at T28d, it was 0.013 ± 0.009; at T3M, it was 0.07 ± 0.08, and at T6M, it was 0.013 ± 0.014 (Figure 1A, lower panel). 

In patients with B-ALL, a peak in the percentage and the absolute number of peripheral CAR-T cells was observed at T14d after the infusion. The percentage of CAR-T cells was at T3d 0.968 ± 2.240; at T7d, it was 2.252 ± 2.017; at T14d, it was 12.667 ± 20.129; at T28d, it was 2.943 ± 6.911; at T3M, it was 0.652 ± 1.118; and at T6M, the number was 0.110 ± 0.096 (Figure 1B, upper panel). The same trend was observed for the absolute number of CAR-T cells × 10^9^/L. At T3d, they were 0.018 ± 0.055; at T7d, they were 0.017 ± 0.026; at T14d, they were 0.098 ± 0.250; at T28d, they were 0.010 ± 0.017; at T3M, they were 0.003 ± 0.04; and at T6M, they were 0.002 ± 0.001 (Figure 1B, lower panel).

Longitudinal CAR-T-cell monitoring results for each DLBCL and B-ALL patient, both as percentages and absolute numbers, are reported in Supplementary Figure S4. A B-cell aplasia was observed in 80.0% (4/5) of DLBCL and 83.3% (5/6) of B-ALL patients in CR at 3 months. The mean absolute counts and percentages of peripheral B cells from DLBCL and B-ALL patients at the different time points studied (T0-T6M) are reported in Supplementary Figure S5.

### 3.3. CD3, CD3/CD4, CD3/CD8, and Treg Peripheral T-Cell Subset Monitoring

**Figure 2:**
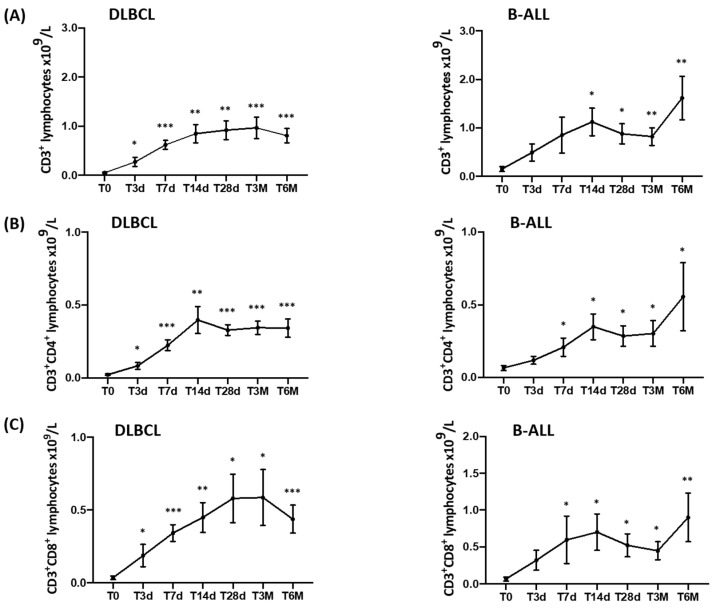
In vivo longitudinal monitoring of peripheral blood (PB) CD3^+^, CD3^+^CD4^+^, and CD3^+^CD8^+^ lymphocytes at different time points before and after CAR-T-cell infusion. Patients’ PB samples were collected prior to the CAR-T-cell infusion (T0) and after 3, 7, 14, and 28 days, as well as 3 and 6 months. Data are reported as means ± standard errors of the absolute number of CD3^+^ (**A**), CD3^+^CD4^+^ (**B**), and CD3^+^CD8^+^ (**C**) lymphocytes in DLBCL (left panel) and B-ALL patients (right panel). Significant differences are calculated compared with T0 and are indicated as *** *p* < 0.001, ** *p* < 0.01, * *p* < 0.05. PB CD3^+^, CD3^+^CD4^+^, and CD3^+^CD8^+^ lymphocytes were gated within the total CD45^+^ leukocyte population.

A constant increase in CD3, CD3/CD4, and CD3/CD8 lymphocytes was observed in both diseases. This increase became significant from the early days after the infusion of CAR-T cells and persisted or increased up to 6 months after the infusion of CAR-T cells (Figure 2).

In DLBCL patients, the number of CD3 lymphocytes at the time of the CAR-T-cell infusion (T0) was 0.052 ± 0.043 × 10^9^/L and constantly increased at the subsequent study points compared with T0 (Figure 2A, left panel). More specifically, at T3d, the number of CD3 cells was 0.296 ± 0.3367 (*p* < 0.05); at T7d, it was 0.618 ± 0.315 (*p* < 0.0001); at T14d, it was 0.846 ± 0.667 (*p* < 0.001); at T28d, it was 0.918 ± 0.692 (*p* < 0.001); at T3M, it was 0.966 ± 0.620 (*p* < 0.0001); and at T6M, it was 0.807 ± 0.354 (*p* < 0.0001).

This increase was also observed for peripheral CD3/CD4 (×10^9^/L) cells. At T0, CD3/CD4 cells were 0.022 ± 0.020 and increased to 0.090 ± 0.082 at T3d (*p* < 0.01), to 0.224 ± 0.127 at T7d (*p* < 0.0001), to 0.397 ± 0.334 at T14d (*p* < 0.001), to 0.328 ± 0.132 at T28d (*p* < 0.0001), to 0.344 ± 0.129 at T3M (*p* < 0.0001), and to 0.342 ± 0.153 at T6M (*p* < 0.0001) (Figure 2B, left panel).

CD3/CD8 lymphocytes from DLBCL patients also increased during the observation period. At T0, they were 0.034 ± 0.041; at T3d, they were 0.204 ± 0.270 (*p* < 0.05); at T7d, they were 0.342 ± 0.200 (*p* < 0.0001); at T14d, they were 0.449 ± 0.369 (*p* < 0.001); at T28d, they were 0.580 ± 0.604 (*p* < 0.01); at T3M, they were 0.586 ± 0.542 (*p* < 0.01); and at T6M, they were 0.437 ± 0.236 (*p* < 0.0001) (Figure 2C, left panel).

In B-ALL patients, the number of circulating CD3 cells (×10^9^/L) significantly increased from T14d, and the highest values were observed at T6M. CD3 cells were 0.151 ± 0.164 at T0, 0.492 ± 0.588 at T3d (NS), 0.853 ± 1.232 at T7d (NS), 1.123 ± 0.986 at T14d (*p* < 0.01), 0.878 ± 0.716 at T28d (*p* < 0.01), 0.820 ± 0.517 at T3M (*p* < 0.001) and 1.618 ± 0.899 at T6M (*p* < 0.001) (Figure 2A, right panel).

A similar pattern was observed for the absolute number (×10^9^/L) of circulating CD3/CD4 cells from B-ALL patients. At T0, they were 0.066 ± 0.057; at T3d, they were 0.119 ± 0.091 (NS); at T7d, they were 0.209 ± 0.207 (*p* < 0.05); at T14d, they were 0.349 ± 0.308 (*p* < 0.01); at T28d, they were 0.286 ± 0.245 (*p* < 0.01); at T3M, they were 0.303 ± 0.245 (*p* < 0.01); and at T6M, they were 0.556 ± 0.466 (*p* < 0.01) (Figure 2B, right panel).

Moreover, peripheral CD3/CD8 cells also increased over time. At T0, they were 0.065 ± 0.082; at T3d, they were 0.319 ± 0.449 (NS); at T7d, they were 0.596 ± 1.069 (NS); at T14d, they were 0.700 ± 0.859 (*p* < 0.05); at T28d, they were 0.523 ± 0.537 (*p* < 0.01); at T3M, they were 0.448 ± 0.344 (*p* < 0.01); and at T6M, they were 0.901 ± 0.661 (*p* < 0.001) (Figure 2C, right panel).

**Figure 3:**
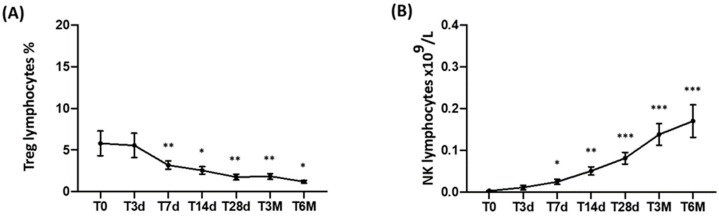
In vivo longitudinal monitoring of peripheral blood (PB) Treg and NK lymphocytes at different time points before and after CAR-T-cell infusion. PB samples from patients admitted to the Sapienza University of Rome (13 DLBCL and 2 B-ALL) were collected prior to CAR-T-cell infusion (T0) and after 3, 7, 14, and 28 days, as well as 3 and 6 months. Data are reported as means ± standard errors of the percentage of Treg (**A**) and of the absolute number of NK (**B**) lymphocytes. Significant differences are calculated compared with T0 and are indicated as *** *p* < 0.001, ** *p* < 0.01, and * *p* < 0.05. PB Treg and NK lymphocytes were gated within the total CD45^+^ leukocyte population.

Treg cells were studied in nine DLBCL patients. The percentage of Treg cells showed a significant decrease over time, being 5.81 ± 4.45% at T0, 5.55 ± 4.12% (NS) at T3d, 3.19 ± 1.48% (NS) at T7d, 2.56 ± 1.28 (NS) at T14d, 1.76 ± 0.97% (*p* < 0.05) at T28d, 1.85 ± 0.81% at T3M (*p* = 0.05), and 1.20 ± 0.35 at T6M (*p* < 0.05) (Figure 3A). 

Data of CD3, CD3/CD4, and CD3/CD8, as well as Treg cell monitoring data at each time point of the study, are reported in Supplementary Table S2.

### 3.4. NK-Cell Monitoring

In a subgroup of 15 patients (13 DLBCL and 2 B-ALL) treated at the Hematology Center in Rome, the circulating NK cells (CD3^−^/CD56^+^/CD16^+^), characterized using flow cytometry, were evaluated as absolute number × 10^9^/L. After CAR-T-cell infusion, NK cells significantly increased over time: at T0, they were 0.003 ± 0.004; at T3d, they were 0.012 ± 0.018 (NS); at T7d, they were 0.025 ± 0.022 (*p* < 0.01); at T14d, they were 0.051 ± 0.035 (*p* < 0.001); at T28d, they were 0.081 ± 0.050 (*p* < 0.0001); at T3M, they were 0.138 ± 0.074 (*p* < 0.0001); and at T6M, they were 0.170 ± 0.095 (*p* < 0.0001) (Figure 3B).

### 3.5. Immunomodulant Cytokine Production Using Stimulated Lymphocyte Population

**Figure 4:**
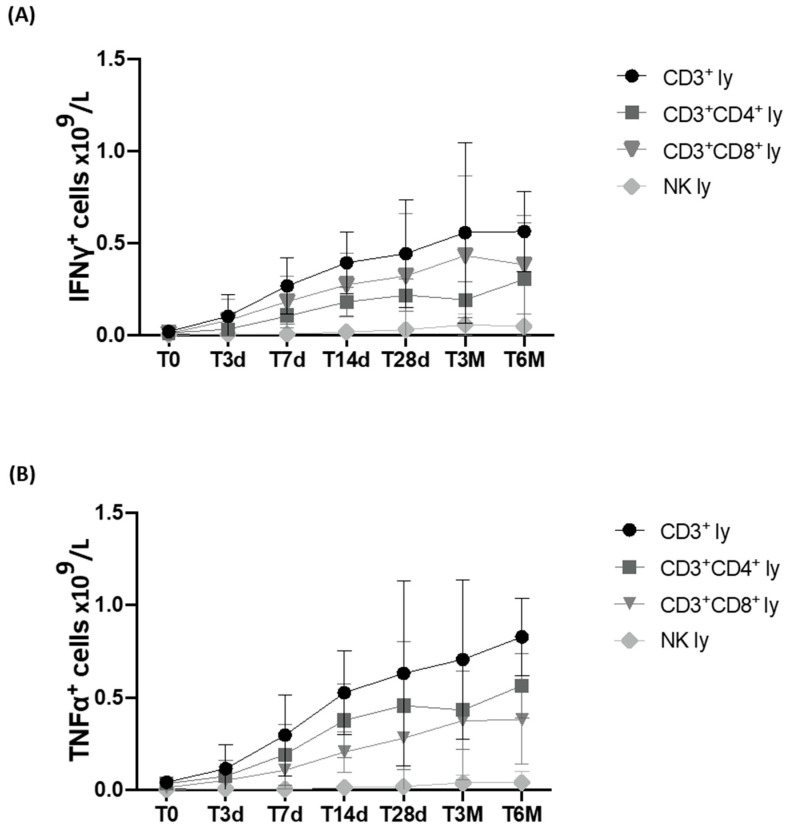
Longitudinal analysis of the absolute number of IFNγ and TNFα producing CD3^+^, CD3^+^CD4^+^, CD3^+^, and CD8^+^ and NK lymphocytes at different time points before and after CAR-T-cell infusion. Peripheral blood (PB) samples from patients enrolled at Sapienza University of Rome (13 DLBCL and 2 B-ALL) were collected prior to CAR-T-cell infusion (T0) and after 3, 7, 14, and 28 days, as well as 3 and 6 months, and activated in vitro to evaluate IFNγ (**A**) and TNFα (**B**) production. Data are reported as means ± standard errors. IFNγ and TNFα producing CD3^+^, CD3^+^CD4^+^, CD3^+^, and CD8^+^ and NK lymphocytes were gated within the total lymphocyte population identified through its forward and side scatter density properties.

In the same 15 patients, the ability of the lymphocyte populations to produce IFNγ and TNFα, which are the cytokines capable of inducing an immune response, was evaluated after CAR-T-cell infusion. A constant increase in intracellular production was observed in CD3, CD3/CD4, and CD3/CD8 cells, which became highly significant already at T3d for IFNγ (*p* < 0.05) and at T7d for TNFα (*p* < 0.005), and increased further at later time points (Figure 4A,B). 

After CAR-T-cell infusion, an increase was also observed in the number of NK cells capable of producing IFNγ and TNFα, which was already significant at T28d and T14d, respectively.

The data of the absolute number of producing cells and of the significance levels reached are presented in Supplementary Table S3.

### 3.6. Plasma Cytokine Levels

Finally, in 15 patients (13 DLBCL and 2 B-ALL), plasma cytokine levels were measured before and after CAR-T-cell infusion. An increase in plasma concentration was observed only for IL-6, IL-8, and IL-10 cytokines in 5/15 patients (Supplementary Figure S6). In contrast, an increase in IL-1β, IL-2, IL-4, IL-12p70, IL-17A, IFNγ, and TNFα cytokines was never observed.

The cytokine plasma levels were correlated with the occurrence or not of a CRS. Indeed, a correlation was observed between the increase in the above-mentioned cytokines and the presence of a clinical CRS. In particular, the levels of IL-6 seemed to be the most sensitive marker in view of an evident increase in plasmatic IL-6 observed in five patients (N° 1, 4, 8, 11, and 15; Supplementary Table S1A) who witnessed a clinical CRS following CAR-T-cell infusion. In the same patients, an increase, although less evident, in plasmatic IL-8 and IL-10 was also observed. By contrast, a lower increase in the same cytokines was observed in the 10/15 patients who did not develop a CRS.

## 4. Discussion

This study aimed to investigate the effect of CAR-T-cell administration in the control of R/R DLBCL and B-ALL and highlight the immunomodulatory effects induced on the host immune system of the treated patients over time in vivo. The results obtained (i) confirmed the ability of tisagenlecleucel to control the disease at least for a limited period of time; (ii) showed that many patients who subsequently relapsed could undergo further treatment; (iii) showed that the in vivo release of cytokines, particularly IL-6, was associated with a clinical CRS; (iv) documented a significant increase in CD3^+^, CD4^+^, CD8^+^, and NK cells over time; (v) indicated that immune modulation was associated with a decrease in the percent of Treg cells and (vi) an increased capacity of T lymphocytes to produce IFNγ and TNFα. 

It is well documented that CAR-T cells represent eligible patients with R/R CD19^+^ B-lineage ALL and DBCL who lack other therapeutic options and an opportunity to obtain a further clinical response and a potential cure [20,21]. This is also what we observed in this study; in fact, most patients showed a rapid response to tisagenlecleucel: 84.6% in DLBCL and 91.7% in B-ALL. In addition, many patients (9/13 DLBCL and 5/12 B-ALL) who relapsed clinically or molecularly (B-ALL) after receiving CAR-T cells could undergo further treatment that could not be offered at the time of starting CAR-T-cell treatment. This allowed for a median OS of 12 months for DLBCL patients and 20 months for B-ALL patients from the time of enrollment to CAR-T-cell therapy. This is a positive outcome in view of the clinical situation of the patients at the time of their entry into the CAR-T-cell program [22,23,24]. Approximately 50% of the patients suffered from a clinical CRS following the CAR-T-cell infusion. We monitored the circulating plasma levels of different cytokines and observed an increase mainly in IL-6 levels, more evident in patients who witnessed a clinical CRS. 

Similar to previous studies [25,26], we also observed an in vivo increase in the infused CAR-T cells in the two groups of patients, albeit with different dynamics. Compared with T0, in patients with DLBCL, we observed an increase in the percentage and absolute number of CAR-T cells between the 3rd and 7th day after the infusion. In patients with B-ALL, the CAR-T-cell peak, both as a percentage and absolute number, was instead recorded on day 14 after the infusion.

While some studies have focused on the characterization of CAR-T-infused cells [27,28], little is known about the in vivo effects of this treatment on the patient’s immune lymphocyte populations. Our study demonstrates that CAR-T cells are capable of inducing a bystander effect on the lymphoid populations of patients evaluated from the time of the infusion (T0) up to six months (T6M) after the treatment. We could in fact document a constant, prolonged, and significant increase in CD3^+^, CD3/CD4^+^, and CD3/CD8^+^, as well as in the NK-cell population (Figure 2 and Figure 3). Even more importantly, we could confirm the ability of T lymphocytes to produce IFNγ and TNFα (Figure 4), cytokines that are essential in the process of recognition and anti-tumor cytotoxic capacity [29,30]. Finally, the percentage of Tregs progressively and significantly decreased over time. 

To the best of our knowledge, these are the first data showing that the infusion of CAR-T cells in patients with DLBCL and B-ALL is capable of exerting a marked and long-lasting modulation of the host immune system, in terms of T- and NK-cell expansion and cytokine release. So far, we had indirect evidence from preclinical models that pointed towards the possible crosstalk between CAR-T cells and the immune system. In a mouse B-cell lymphoma model, Boulch et al. [15] reported the impact of CD4 and CD8 CAR-T cells on the host immune system and the possible role of cytokines released in vivo, underlining the importance of the crosstalk between CAR-T cells and the tumor microenvironment in order to enable an optimal anti-tumor CAR-T-cell efficacy. Based on the data obtained in a syngeneic mouse glioblastoma, it has also been reported that CAR-T cells have the potential to establish crosstalk with the tumor microenvironment, essential to promoting endogenous anti-tumor immunity involving the in vivo production of IFNγ [16]. In addition, Chen et al. [22], through the histopathological examination of the DLBCL tumor microenvironment in patients treated with the anti-CD19 CAR-T-cell-product axicabtagene ciloleucel, demonstrated the presence of high levels of activated non-CAR-T cells expressing Ki-67, IFNγ, granzyme B, and/or PD-1. These non-CAR-T cells were also the exclusive source of IL-6, the most crucial cytokine associated with the CRS. The authors suggested a role for CAR-T cells in activating non-CAR immune cells within the tumor microenvironment, enhancing the anti-tumoral cytotoxicity as well as the risk of immune-mediated adverse effects. Alizadeh et al. [16] reported that CAR-T cells can boost the activity of resident myeloid cells and endogenous T cells, emphasizing the role of both innate and adaptive host immunity for the CAR-T-cell therapy of solid tumors. Taken together, these results indicate that the role of CAR-T cells appears to extend beyond their capacity to kill neoplastic cells, showing that in DLBCL and B-ALL, they are also capable of exerting a marked modulation and activation of the patient’s immune compartment that persists over time. It is likely that this in vivo host immune modulation may play a role in the long-term control of the disease, at a time when CAR-T cells may be bearably found in treated patients [22,31]. This could also help to explain how patients relapsing after CAR-T cells may become amenable to treatment strategies that could not be considered earlier, including allogeneic stem cell transplantation. These observations need further confirmation in larger series of CAR-T-cell-treated patients but contribute to a further understanding of the complex field of immunotherapy and intercellular crosstalk. These findings are reminiscent of what was recently observed in adult Ph^+^ ALL patients treated with the bispecific monoclonal antibody blinatumomab following dasatinib administration in induction. Blinatumomab induced a marked host immune modulation that presumably plays a role in controlling the disease [32,33,34].

## 5. Conclusions

In summary, our results suggest that in patients with DLBCL and B-ALL, the administration of tisagenlecleucel is capable of inducing a marked and prolonged in vivo reshaping of the host immune system, both in children and adults, and they provide the basis for a better understanding of the immunologic signatures associated with CAR-T-cell therapy efficacy. 

## Data Availability

The data that support the findings of this study are available from the corresponding author upon reasonable request.

## Supplementary Materials

The following supporting information can be downloaded at: https://www.mdpi.com/article/10.3390/cancers15092411/s1, Table S1: Demographic and clinical characteristics and CAR-T-cell dosage of the patients included in the study; Table S2: Longitudinal monitoring of CD3^+^, CD3^+^CD4^+^, CD3^+^CD8^+^, NK lymphocytes, and Treg cells at different time points before and after CAR-T-cell infusion; Table S3: Longitudinal analysis of the absolute number of IFNγ and TNFα producing CD3^+^, CD3^+^CD4^+^, CD3^+^CD8^+^, and NK lymphocytes at different time points after CAR-T-cell infusion. Figure S1: Representative plots of the flow gating strategies to detect CAR-T cells and non-CAR-T lymphocytes within the total CD45^+^ leukocyte population; Figure S2: Longitudinal monitoring of whole-blood count (WBC) and CD3^+^ lymphocytes at different time points before and after infusion; Figure S3: Representative plots of the flow gating strategies to detect IFNγ and TNFα producing CD4, CD8, and NK cells; Figure S4: Longitudinal monitoring of CD19 CAR-T cells at different time points after infusion; Figure S5: Longitudinal monitoring of B lymphocytes at different time points before and after CAR-T-cell infusion; Figure S6: Plasmatic levels of IL-6 (a), IL-8 (b) and IL-10 (c) in DLBCL (n = 13) and B-ALL (n = 2) patients at different time points after CAR-T-cell infusion.
