# Supplementary material for: Long-Term Host Immune Modulation Following Tisagenlecleucel Administration in Patients with Diffuse Large B-Cell Lymphoma and B-Lineage Acute Lymphoblastic Leukemia
Source: Cancers (Basel). 2023 Apr 22;15(9):2411. doi: 10.3390/cancers15092411 (PMC10177375; doi:10.3390/cancers15092411)

Table S1. Demographics, clinical characteristics and CAR-T cell dosage for the patients included in the study. A total of 25 patients were enrolled: 15 patients from the Rome Center (**A**) and 10 patients from the Monza Center (**B**).

(A)

PATIENTS	DISEASE	SEX	AGE	CAR-T cell dosage	CRS (Grade)	FOLLOW-UP (Months)	STATUS Alive (+) Deceased (-)
**#1**	B-ALL	F	15	0.7×10^8	4	3	-
**#2**	DLBCL	F	52	3.5×10^8	–	28	+
**#3**	DLBCL	M	66	2.7×10^8	–	21	+
**#4**	DLBCL	M	49	2.0×10^8	2	11	-
**#5**	B-ALL	M	20	1.1×10^8	–	21	+
**#6**	DLBCL	M	59	2.0×10^8	–	12	-
**#7**	DLBCL	F	52	1.7×10^8	–	17	+
**#8**	DLBCL	M	69	2.8×10^8	2	15	-
**#9**	DLBCL	M	70	2.8×10^8	2	10	-
**#10**	DLBCL	M	43	3.1×10^8	1	11	+
**#11**	DLBCL	F	45	2.1×10^8	2	6	-
**#12**	DLBCL	F	53	4.8×10^8	1	5	-
**#13**	DLBCL	M	48	3.0×10^8	1	3	+
**#14**	DLBCL	F	71	2.1×10^8	1	4	+
**#15**	DLBCL	M	34	3.3×10^8	1	4	-

(B)

PATIENTS	DISEASE	SEX	AGE	CAR-T cell dosage	CRS (Grade)	FOLLOW-UP (Months)	STATUS Alive (+) Deceased (-)
**#1**	B-ALL	F	16	1.4×10^8	4	18	-
**#2**	B-ALL	M	21	1.6×10^6	–	28	+
**#3**	B-ALL	M	8	1.4×10^6	–	27	+
**#4**	B-ALL	F	4	2.9×10^6	1	29	+
**#5**	B-ALL	M	3	1.1×10^6	–	23	+
**#6**	B-ALL	M	9	4.2×10^6	2	20	+
**#7**	B-ALL	M	7	2.9×10^6	1	20	+
**#8**	B-ALL	M	6	3.9×10^6	–	12	-
**#9**	B-ALL	M	4	2.6×10^6	–	18	+
**#10**	B-ALL	F	6	1.6×10^6	–	16	+

Table S2. Longitudinal monitoring of CD3$^+$, CD3$^+$CD4$^+$, CD3$^+$CD8$^+$, NK lymphocytes and Treg cells at different time points before and after CAR-T cell infusion. Patients' PB samples were collected prior to the CAR-T cell infusion (T0) and after 3, 7, 14, 28 days and 3, 6 months. Data are reported as means ± standard errors of the absolute numbers of CD3$^+$, CD3$^+$CD4$^+$ and CD3$^+$CD8$^+$ for both DLBCL and B-ALL patients, and the absolute numbers of NK cells and the percentages of Tregs for DLBCL patients. Significant differences are calculated compared to T0.

	**CD3$^+$ Lymphocytes (x10^9/L)**				**CD3$^+$CD4$^+$ Lymphocytes (x10^9/L)**				**CD3$^+$CD8$^+$ Lymphocytes (x10^9/L)**				**NK Lymphocytes (x10^9/L)**		**Treg (%)**	
	**DLBCL**	*p value*	**B-ALL**	*p value*	**DLBCL**	*p value*	**B-ALL**	*p value*	**DLBCL**	*p value*	**B-ALL**	*p value*	**DLBCL**	*p value*	**DLBCL**	*p value*
**T0**	0.05± 0.01		0.15 ± 0.05		0.02 ± 0.01		0.07 ± 0.02		0.03 ± 0.01		0.07 ± 0.02		0.00 ± 0.00		5.81 ± 1.48	
**T3d**	0.27 ± 0.10	*3.4E-02*	0.49 ± 0.18	*7.9E-02*	0.08 ± 0.02	*2.2E-02*	0.12 ± 0.03	*1.2E-01*	0.19 ± 0.08	*6.1E-02*	0.32 ± 0.14	*8.0E-02*	0.01 ± 0.01	*1.4E-01*	5.55 ± 1.46	*9.0E-01*
**T7d**	0.62 ± 0.09	*3.3E-06*	0.85 ± 0.37	*7.6E-02*	0.22 ± 0.04	*1.8E-05*	0.21 ± 0.06	*3.9E-02*	0.34 ± 0.06	*3.0E-05*	0.60 ± 0.32	*1.2E-01*	0.02 ± 0.01	*2.4E-03*	3.19 ± 0.50	*1.3E-01*
**T14d**	0.85 ± 0.18	*4.3E-04*	1.22 ± 0.29	*1.8E-03*	0.40 ± 0.09	*7.6E-04*	0.38 ± 0.09	*3.1E-03*	0.45 ± 0.10	*7.8E-04*	0.76 ± 0.26	*1.7E-02*	0.05 ± 0.01	*9.7E-05*	2.56 ± 0.45	*6.6E-02*
**T28d**	0.92 ± 0.19	*2.5E-04*	0.95 ± 0.21	*1.5E-03*	0.33 ± 0.04	*4.7E-08*	0.31 ± 0.07	*4.0E-03*	0.58 ± 0.17	*4.8E-03*	0.57 ± 0.16	*6.4E-03*	0.08 ± 0.01	*1.5E-05*	1.76 ± 0.34	*2.4E-02*
**T3M**	0.97 ± 0.22	*6.6E-05*	0.82 ± 0.18	*8.1E-04*	0.34 ± 0.05	*8.2E-08*	0.30 ± 0.09	*6.2E-03*	0.59 ± 0.19	*2.2E-03*	0.45 ± 0.12	*2.3E-03*	0.14 ± 0.03	*4.6E-06*	1.85 ± 0.33	*5.3E-02*
**T6M**	0.81 ± 0.14	*1.2E-06*	1.39 ± 0.42	*5.5E-04*	0.34 ± 0.06	*1.7E-06*	0.51 ± 0.19	*2.5E-03*	0.44 ± 0.10	*2.2E-05*	0.74 ± 0.30	*4.0E-03*	0.17 ± 0.04	*1.1E-05*	1.20 ± 0.16	*4.2E-02*

Table S3. Longitudinal analysis of the absolute number of IFNγ and TNFα producing $CD3^+$, $CD3^+CD4^+$, $CD3^+CD8^+$ and NK lymphocytes at different time points after CAR-T cell infusion. Peripheral blood (PB) samples from patients enrolled at Sapienza University of Rome (13 DLBCL and 2 B-ALL) were collected prior to CAR-T cell infusion (T0) and after 3, 7, 14, 28 days and 3, 6 months and activated *in vitro* to evaluate IFNγ and TNFα production. Data are reported as means ± standard errors. Significant differences are calculated respect to T0.

	$CD3^+$ Lymphocytes ($x10^9/L$)				$CD3^+CD4^+$ Lymphocytes ($x10^9/L$)				$CD3^+CD8^+$ Lymphocytes ($x10^9/L$)				NK Lymphocytes ($x10^9/L$)			
	**IFNγ**	*p value*	**TNFα**	*p value*	**IFNγ**	*p value*	**TNFα**	*p value*	**IFNγ**	*p value*	**TNFα**	*p value*	**IFNγ**	*p value*	**TNFα**	*p value*
**T0**	0.02 ± 0.02		0.04 ± 0.03		0.01 ± 0.01		0.03 ± 0.03		0.01 ± 0.01		0.01 ± 0.01		0.00 ± 0.00		0.00 ± 0.00	
**T3d**	0.10 ± 0.12	*2.8E-02*	0.11 ± 0.13	*6.5E-02*	0.03 ± 0.03	*3.2E-02*	0.07 ± 0.09	*1.4E-01*	0.08 ± 0.11	*4.6E-02*	0.05 ± 0.08	*9.5E-02*	0.01 ± 0.01	*3.3E-02*	0.00 ± 0.01	*8.5E-01*
**T7d**	0.27 ± 0.15	*1.8E-05*	0.30 ± 0.22	*6.4E-04*	0.10 ± 0.04	*5.9E-07*	0.19 ± 0.17	*3.6E-03*	0.18 ± 0.14	*3.3E-04*	0.11 ± 0.10	*4.4E-03*	0.01 ± 0.00	*8.0E-03*	0.00 ± 0.00	*4.5E-02*
**T14d**	0.39 ± 0.17	*1.7E-07*	0.53 ± 0.23	*3.4E-07*	0.18 ± 0.08	*3.6E-07*	0.38 ± 0.20	*6.6E-06*	0.27 ± 0.17	*2.1E-05*	0.20 ± 0.11	*4.1E-06*	0.02 ± 0.01	*8.6E-04*	0.01 ± 0.01	*2.4E-03*
**T28d**	0.44 ± 0.29	*4.7E-05*	0.63 ± 0.50	*5.1E-04*	0.22 ± 0.09	*4.8E-08*	0.46 ± 0.35	*3.7E-04*	0.32 ± 0.34	*4.7E-03*	0.28 ± 0.36	*1.7E-02*	0.03 ± 0.03	*9.5E-04*	0.02 ± 0.02	*5.7E-03*
**T3M**	0.56 ± 0.49	*1.2E-03*	0.71 ± 0.43	*3.8E-05*	0.19 ± 0.10	*4.0E-06*	0.43 ± 0.21	*3.9E-06*	0.43 ± 0.43	*2.9E-03*	0.37 ± 0.32	*9.3E-04*	0.05 ± 0.06	*5.4E-03*	0.04 ± 0.04	*1.1E-02*
**T6M**	0.56 ± 0.22	*2.5E-07*	0.83 ± 0.21	*1.8E-09*	0.30 ± 0.30	*3.0E-03*	0.56 ± 0.18	*3.1E-08*	0.38 ± 0.27	*1.4E-04*	0.38 ± 0.24	*5.9E-05*	0.05 ± 0.07	*2.2E-02*	0.04 ± 0.06	*3.8E-02*

Figure S1. Representative plots of the flow gating strategies to detect CAR-T cells and non-CAR-T lymphocytes within the total $CD45^{+}$ leukocyte population. (**A**) $CD3^{+}CAR^{+}$ cells are depicted in red, $CD3^{+}$ non-CAR-T lymphocytes in blue, CD4 and CD8 within the $CD3^{+}$ non-CAR-T lymphocytes subset in green and in violet, respectively, and $CD3^{-}CD56^{+}$ NK cells in cyan. (**B**) Tregs were identified as $CD25^{+++}CD127^{-}$ cells within the $CD45^{+}CD3^{+}CD4^{+}$ subset.

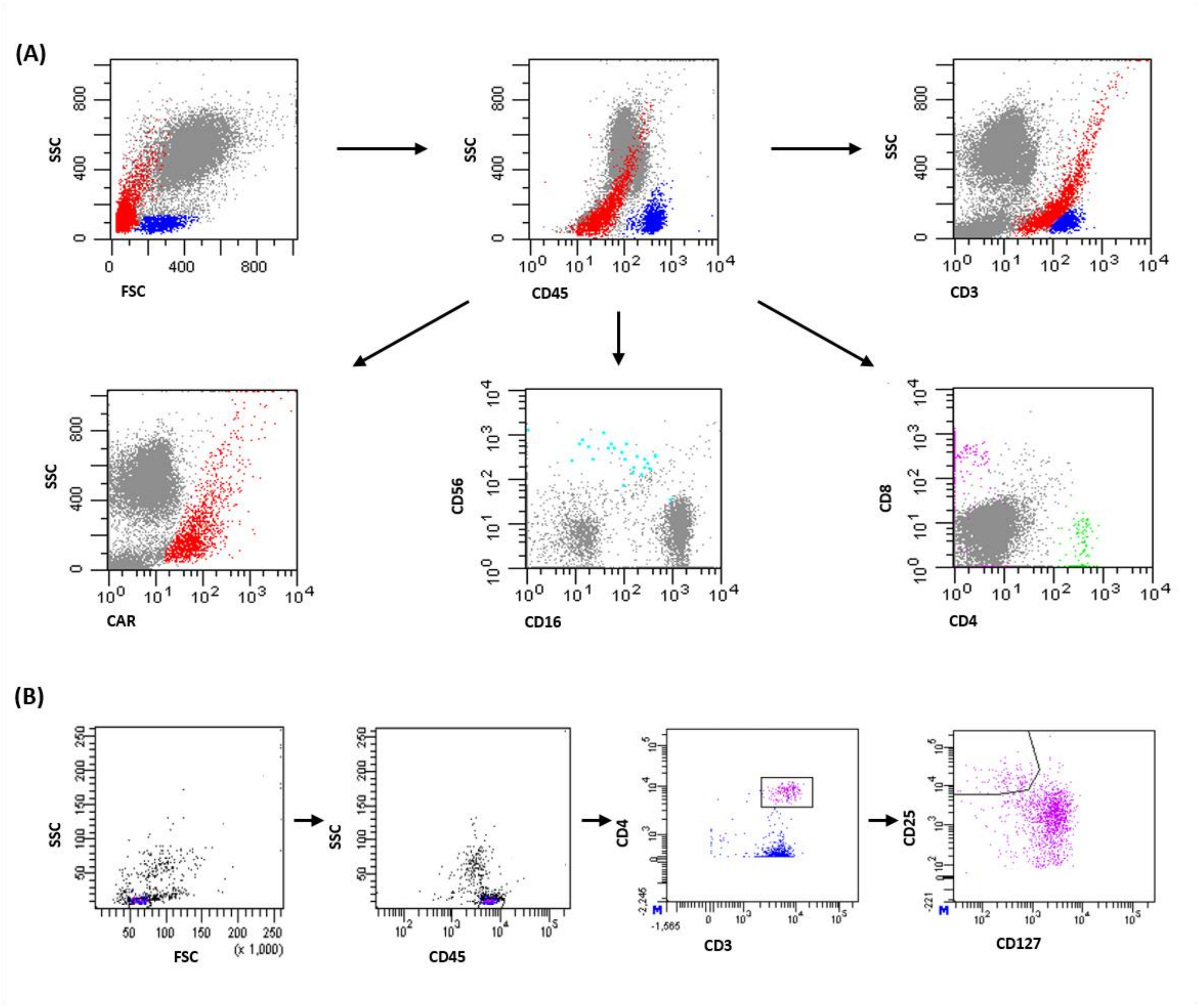

Figure S2. Longitudinal monitoring of whole blood count (WBC) and $CD3^+$ lymphocytes at different time points before and after infusion. Data are reported as means ± standard errors of absolute WBC (**A**) and $CD3^+$ lymphocytes percentage (**B**) in DLBCL (left panel) and B-ALL patients (right panel) up to 6 months from infusion.

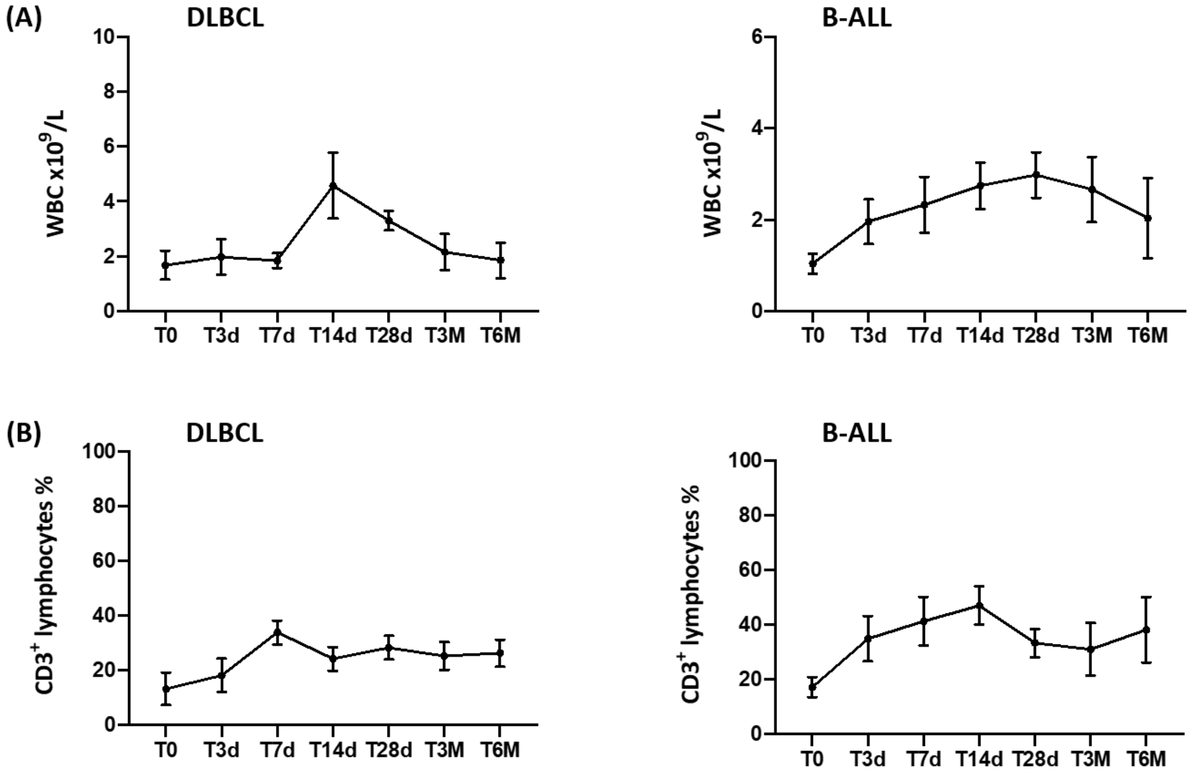

Figure S3. Representative plots of the flow gating strategies to detect IFNγ and TNFα producing CD4, CD8 and NK lymphocytes. Cytokines producing $CD3^{+}CD56^{-}$ T cells and $CD3^{-}CD56^{+}$ NK were gated within the total lymphocyte population identified through its forward and side scatter density properties. CD4 and CD8 lymphocytes were identified as $CD3^{+}CD4^{+}$ and $CD3^{+}CD4^{-}$ cells within the $CD3^{+}CD56^{-}$ T cells subset. IFNγ and TNFα producing CD4 cells are depicted in orange, IFNγ and TNFα producing CD8 cells in blue and IFNγ and TNFα producing NK cells in violet.

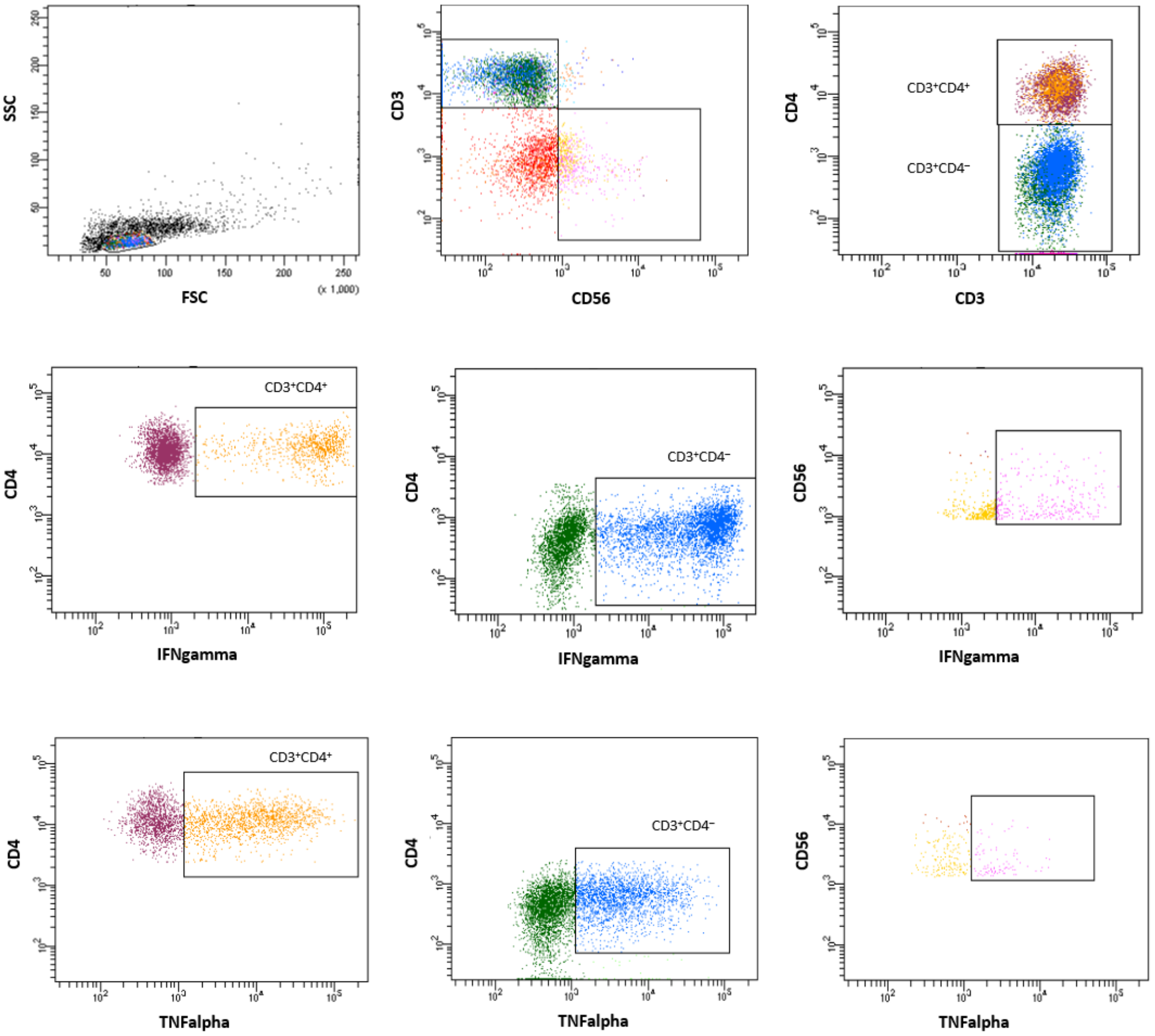

Figure S4. Longitudinal monitoring of CD19 CAR-T cells at different time points after infusion. CAR-T cells were detected by flow cytometry up to 6 months from infusion. Data are reported as individual values of the percentage (upper panel) and absolute number (lower panel) of CAR^{+} cells in DLBCL (**A**) and B-ALL patients (**B**).

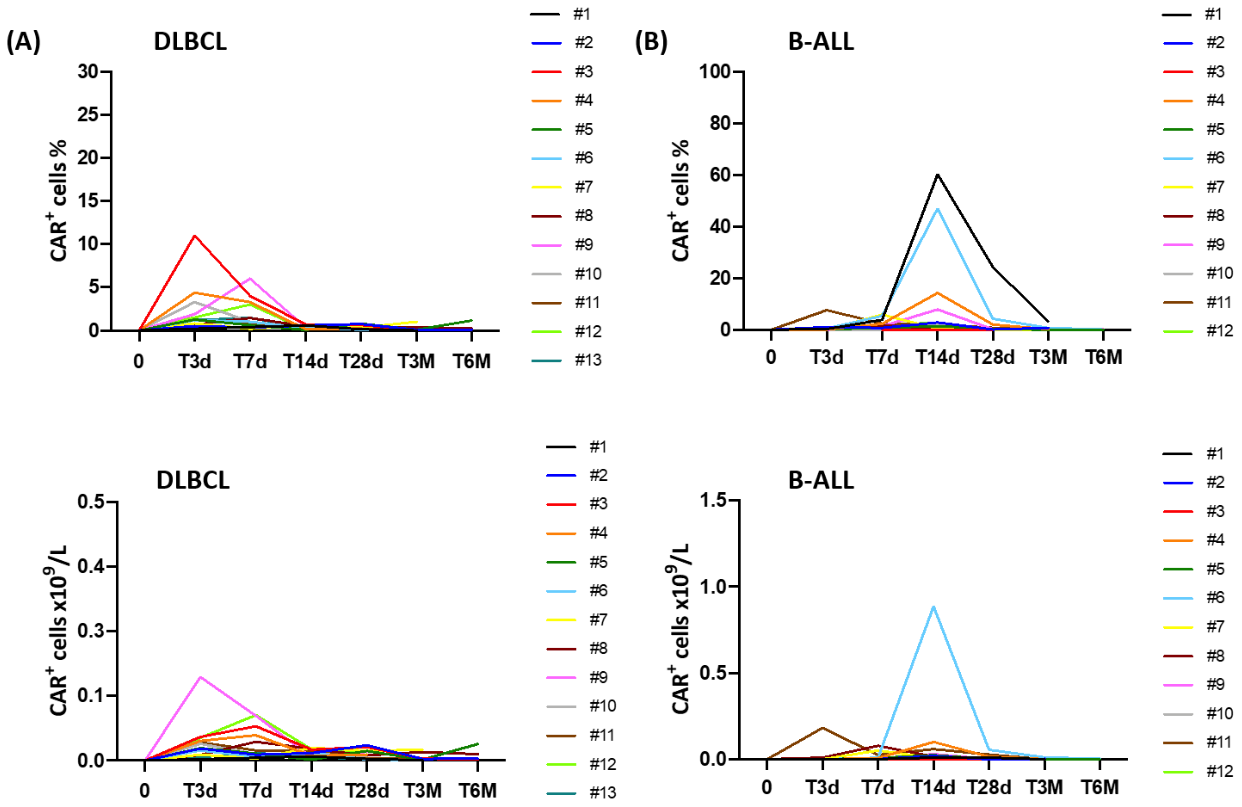

Figure S5. Longitudinal monitoring of B lymphocytes at different time points before and after CAR-T cell infusion. B lymphocytes were detected by flow cytometry up to 6 months from infusion. Data are reported as means ± standard errors of the percentage (**A**) and absolute number (**B**) of $CD19^+$ B cells in DLBCL and B-ALL patients.

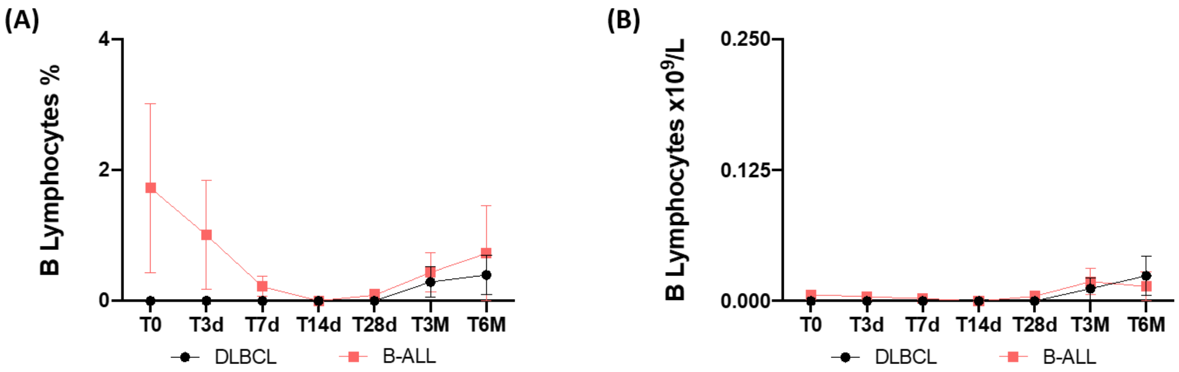

Figure S6. Plasmatic levels of IL-6 (a), IL-8 (b) and IL-10 (c) in DLBCL (n=13) and B-ALL (n=2) patients at different time points after CAR-T cell infusion. Plasma was collected from the PB of patients enrolled at Sapienza University of Rome (13 DLBCL and 2 B-ALL) prior to CAR-T cell infusion (T0) and after 3, 7, 14, 28 days and 3, 6 months. Data are reported as logarithmic cytokine concentration (pg/ml).

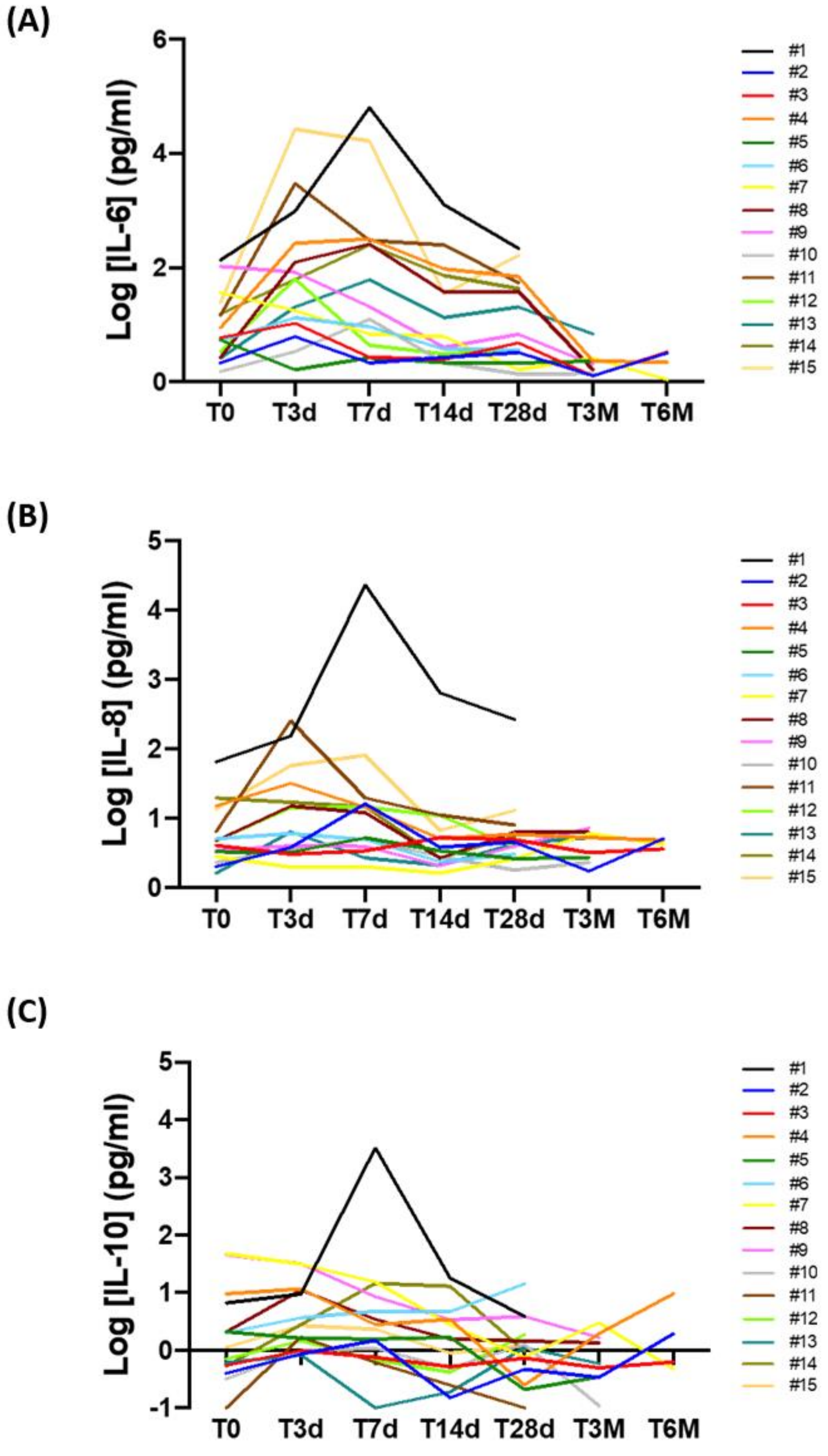

Supplement: Supplementary file 1 [file cancers-15-02411-s001.zip › cancers-2239379-supplementary.pdf]
